# Approaches to eradication of latent HIV infection

**DOI:** 10.1186/1742-4690-10-S1-O21

**Published:** 2013-09-19

**Authors:** David Margolis

**Affiliations:** 1 University of North Carolina at Chapel Hill, USA

## 

A serious effort has begun to develop therapies that may be capable of eradicating established HIV infection in man. Because of the biological complexity of HIV infection that persists despite potent antiretroviral therapy, it is widely believed that if such therapies can be developed they will involve complex, multimodality approaches. An inhibitor of histone deacetylase has been demonstrated to disrupt latency in man, and new histone deacetylase inhibitors have been identified. Other potential targets, such as histone methyltransferase, protein kinase C, and BRD4, have been recently studied. Model systems, both in primary cells and in animal models, are beginning to be validated. In the clinic, immune-based therapies to aid in the clearance of persistent infection are also being tested. It is too early to know what combination eradication therapies for HIV infection will look like in the future, but candidate therapies and model systems to perform preclinical validation are beginning to take shape.

